# Supplementary material for: A single resistance factor to solve vineyard degeneration due to grapevine fanleaf virus
Source: Commun Biol. 2021 May 28;4:637. doi: 10.1038/s42003-021-02164-4 (PMC8163887; doi:10.1038/s42003-021-02164-4)

Supplementary Information

Supplementary Table 1. Infection tests of 14 descendants of a Riesling x Gewurztraminer cross and the Kober 5BB control. For each descendant, 5 to 7 cuttings obtained by vegetative propagation are analysed after infection. The number of grapevines infected by GFLV is revealed by DAS-ELISA on leaves following *X. index*-mediated transmission performed through 36 months of greenhouse cultivation in containers filled with vineyard soil naturally infested by viruliferous nematodes. For each of the descendants, the infection rate, i.e. the ratio of the number of infected cuttings to the number of studied cuttings, is calculated.

Genotype	Number of tested plants	% of GFLV-positive plants	Status
Kober 5BB	7	100%	Susceptible
RsxGw-0018E	6	100%	Susceptible
RsxGw-0013D	5	100%	Susceptible
RsxGw-0032D	6	100%	Susceptible
RsxGw-0071E	6	100%	Susceptible
RsxGw-0045E	5	100%	Susceptible
RsxGw-0302E	6	100%	Susceptible
RsxGw-0203E	6	83%	Susceptible
RsxGw-0069E	5	100%	Susceptible
RsxGw-0016E	5	100%	Susceptible
RsxGw-0075E	6	100%	Susceptible
RsxGw-0027E	6	100%	Susceptible
RsxGw-4076G	5	100%	Susceptible
RsxGw-0015E	5	100%	Susceptible
RsxGw-4095G	6	100%	Susceptible

Supplementary Table 2. List of the previously unpublished markers and their corresponding primers

Marker name	Forward primer	Reverse primer
Chr1_1535	CCATTCTTTTACGCCCTATAC	TAGTTTTACAAGTGCCCCAGT
Chr1_2047	CCTGCACTCATATTAAAGGGT	TCATTGCTCCTAACCATACCT
Chr1_2132	CCATGCAAAGCAATACTGTC	GTGACCCACCTTCACATTTAT
Chr1_2519	TAATCGGTTTGATCGCTAGTC	CCCAATAGCTTGTCCAATTC
Chr1_2869	GGAGGATTATGTGTAGCACTGA	GTAGGAAGAATGACAAAGCCA
Chr1_3175	AAGACCCTGACCTCATCAAA	TCCAAGTGAACCTACAAAACC
VVCS1H024F14R1-1	TCCTGGCTGTCTTCCTTT	CAAGTGGTTGCTCAGGTATC

Supplementary Table 3. Locations and sizes of *V. vinifera* orthologues of recessive genes of resistance to viruses identified in other plant species.

Gene name	Chromosome	Start	End	Strand	Gene id	Annotation	Gene size (bp)	Transcript size (bp)
eIF4E	10	4,024,027	4,029,294	-	GSVIVT01021323001	(2)	5,268	711
eIF(iso)4E	5	9,208,762	9,219,691	-	GSVIVT01027903001	(2)	10,930	573
eIF4G	15	10,927,498	10,942,991	+	VIT_215s0021g00980.4	(1)	15,494	13,287
eIF(iso)4G1	4	6,768,477	6,777,086	-	GSVIVT01035980001	(2)	8,610	2,511
eIF(iso)4G2	11	8,303,024	8,313,124	-	GSVIVT01023638001	(2)	10,101	1,893
PDIL5-1	6	4,016,600	4,032,119	-	GSVIVT01025140001	(2)	15,520	447
cPGK2	19	22,739,784	22,748,143	-	VIT_219s0027g01780	(1)	8,360	1,437
VPS41	6	18,512,705	18,537,533	-	GSVIVT01031443001	(2)	24,829	2,727

(1) https://urgi.versailles.inra.fr/jbrowse/gmod_jbrowse/?data=myData%2FVitisV2%2Fdata&loc=chr19%3A22738141..22750330&tracks=Markers&highlight=

(2) https://wwwdev.genoscope.cns.fr/vitis/

Supplementary Figure 1. Distribution of plants in the container used for testing resistance to grapevine fanleaf disease among 44628 progeny. Each square represents one plant position: resistant 44628 individuals are in green and susceptible 44628 individuals are in red. GFLV was detected in all the susceptible controls (in blue). The dead plants and plants not included in the experimental design are in grey.

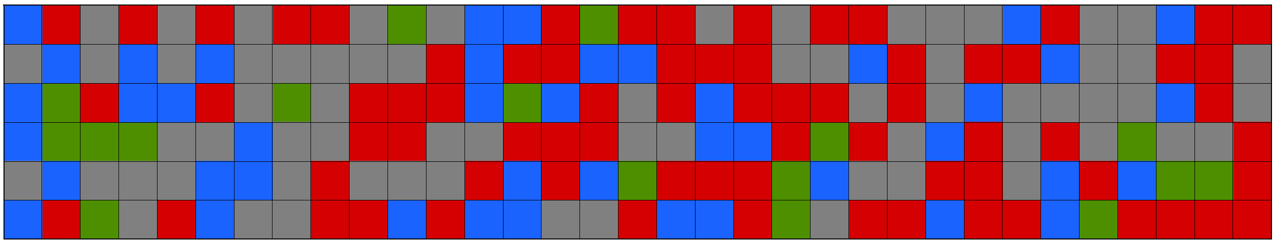

Supplementary Figure 2. Physical position of the SSR markers used to locate the chromosome carrying the putative major factor of resistance, *rgflv1.*

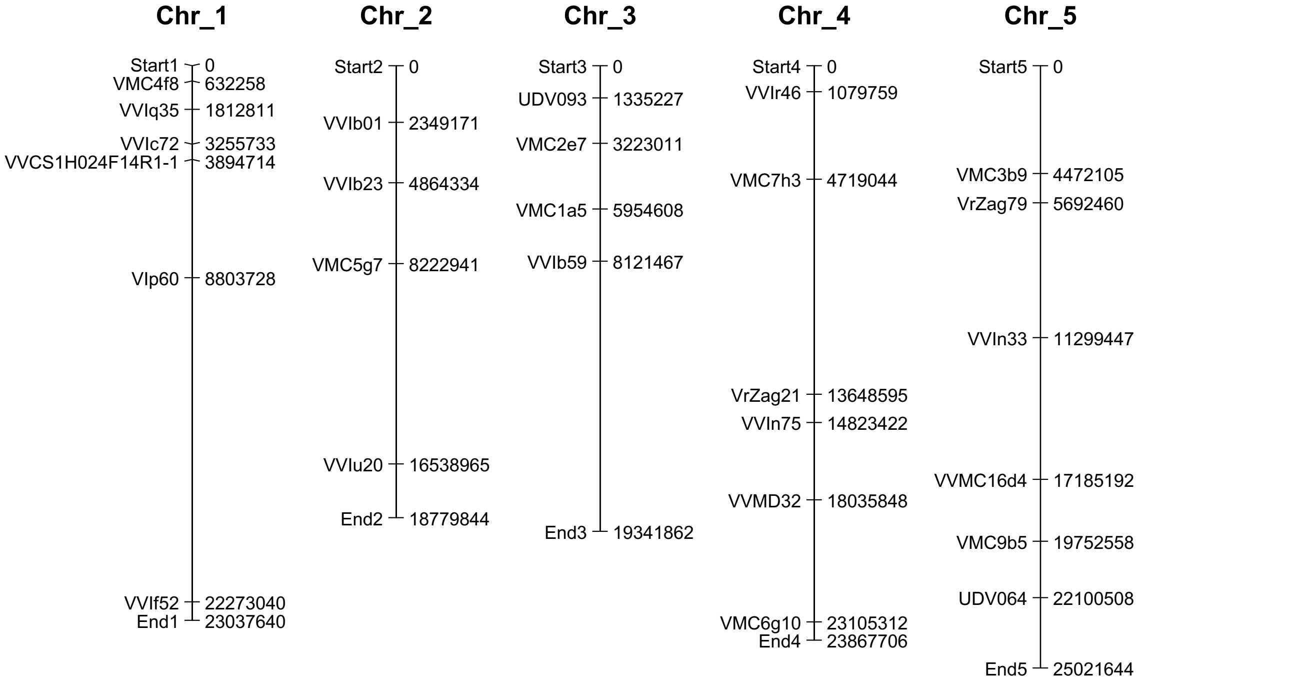

Supplementary Figure 2. Continued

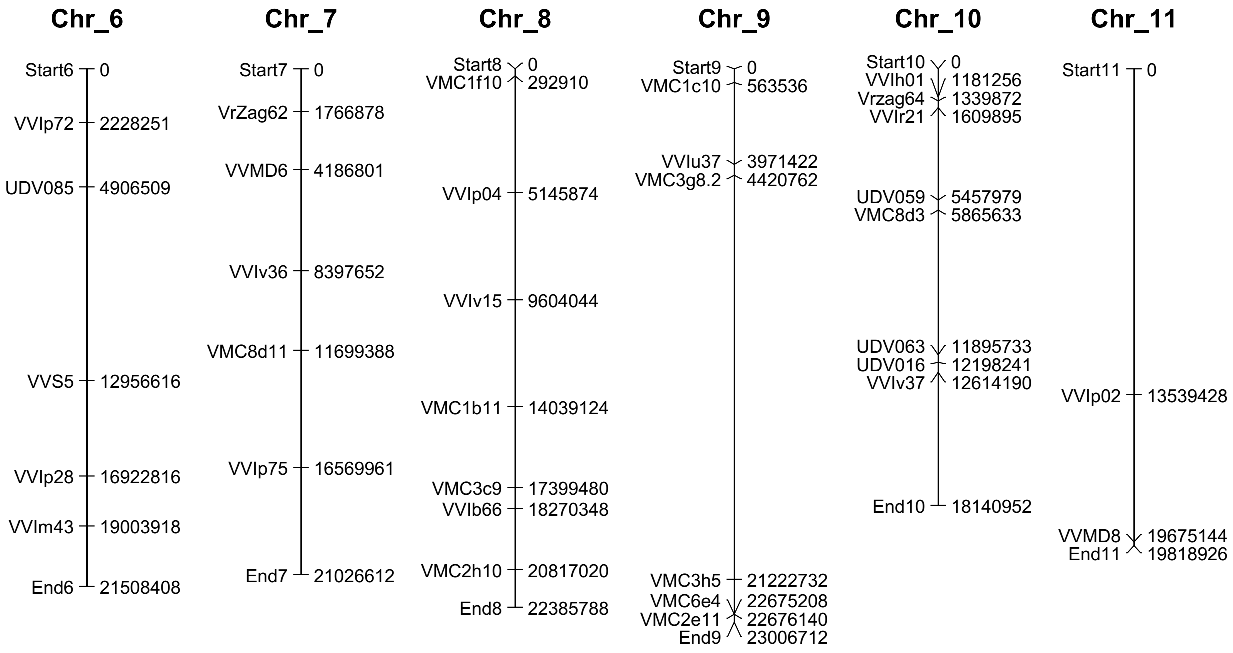

Supplementary Figure 2. Continued

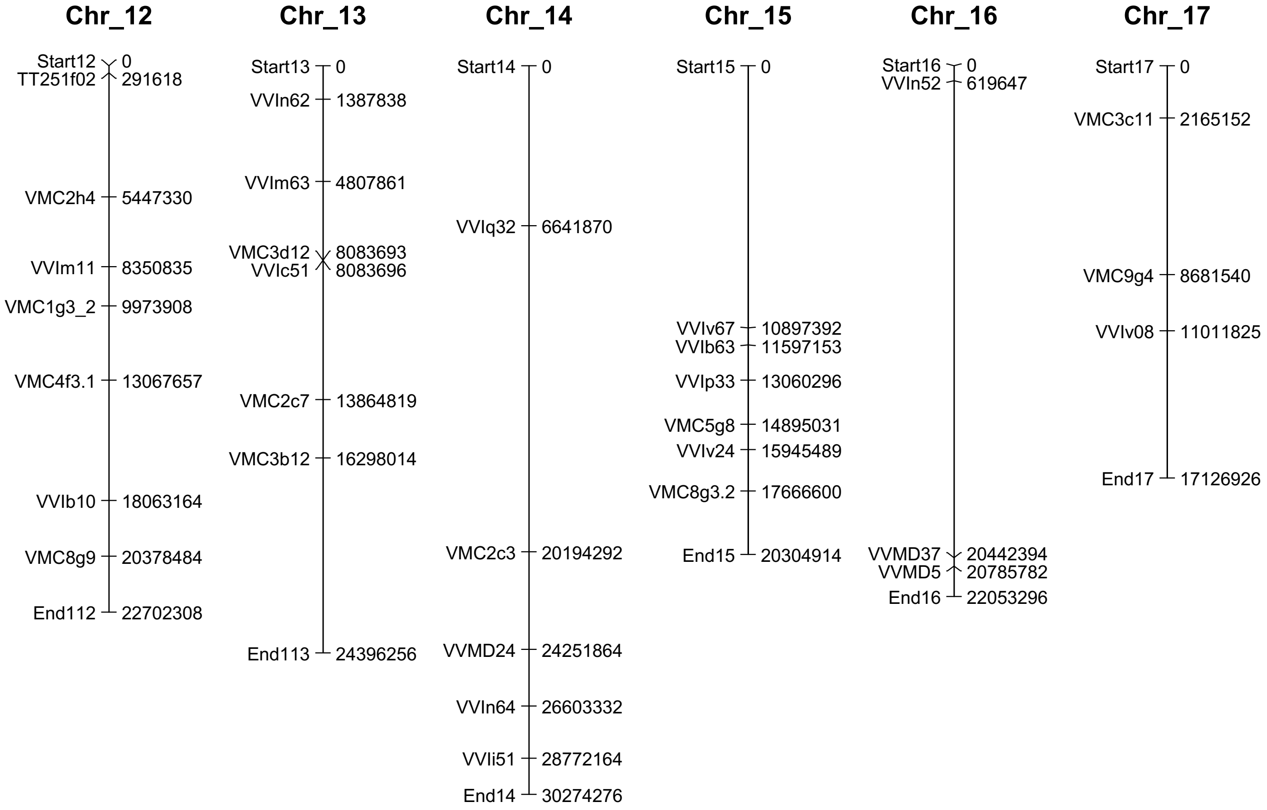

Supplementary Figure 2. Continued

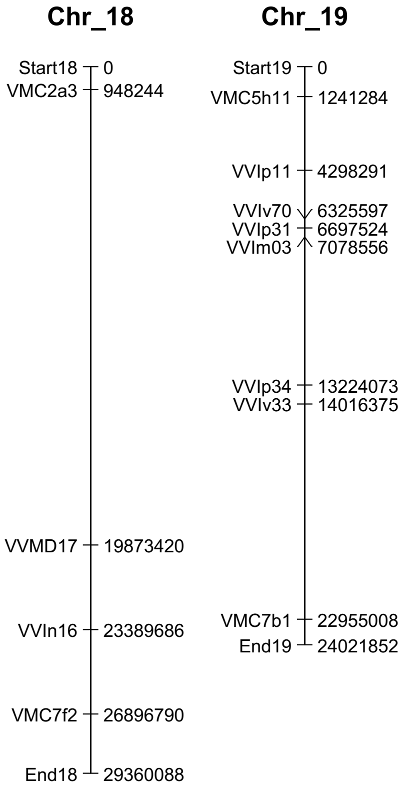

Supplement: Supplementary file 2 — Supplementary Information [file 42003_2021_2164_MOESM2_ESM.pdf]
