# Supplementary material for: A single resistance factor to solve vineyard degeneration due to grapevine fanleaf virus
Source: Commun Biol. 2021 May 28;4:637. doi: 10.1038/s42003-021-02164-4 (PMC8163887; doi:10.1038/s42003-021-02164-4)
Supplement: Supplementary file 3 — Description of Supplementary Files [file 42003_2021_2164_MOESM3_ESM.pdf]

Description of Additional Supplementary Files

File name: Supplementary Data 1.
Description: Maximum ELISA value (max_ELISA) scored over the four years of the experiment in the individuals of 44628 population; max_ELISA is considered not available (NA) when the absence of GFLV (GFLV not detected by DAS-ELISA) could not be shown for at least three years.

File name: Supplementary Data 2.
Description: Marker segregation in resistant and susceptible groups of individuals selected from the 44628 population. Highly significant p-values (<0.0001) and the corresponding markers are in blue.

File name: Supplementary Data 3.
Description: Raw data from genotyping of SSR markers located on chromosome 1 around the *rgflv1* locus for population 44628
