# Supplementary material for: A single resistance factor to solve vineyard degeneration due to grapevine fanleaf virus
Source: Commun Biol. 2021 May 28;4:637. doi: 10.1038/s42003-021-02164-4 (PMC8163887; doi:10.1038/s42003-021-02164-4)
Supplement: Supplementary file 5 — Supplementary Data 2 [file 42003_2021_2164_MOESM5_ESM.pdf]

Supplementary Data 2. Marker segregation in resistant and susceptible groups of individuals selected from the 44628 population. Highly significant p-values (<0.0001) and the corresponding markers are in blue.

			Distribution of the resistant individuals			Distribution of the susceptible individuals						Genotype coding			Genotype of the parent and grand-parents		
**Marker**	**Chromosome**	**Physical position**	**Rhh**	**Rhk**	**Rkk**	**Shh**	**Shk**	**Skk**	**total**	**χ^3**	**p-value**	**hh**	**hk**	**kk**	**0071E**	**Riesling**	**Gewurztraminer**
VMC4f8	1	632 258	0	1	10	3	7	1	22	27.091	0.000055	119	113-119	113	113-119	113-138	119-138
VVIq35	1	1 812 811	0	0	11	4	7	0	22	36.727	0.00001	385	385-389	389	385-389	385-389	385
VVIc72	1	3 255 733	0	1	9	4	6	0	20	26.2	0.000082	250	250-256	256	250-256	250-256	250
VVCS1H024F1	1	3 894 714	0	1	10	4	7	0	22	29.273	0.000021	304	298-304	298	298-304	NA	304
VIp60	1	8 803 728	3	5	3	5	4	2	22	2.545	0.770459	304	304-322	322	304-322	304-322	304-318
VVIf52	1	22 273 039	3	5	3	3	5	3	22	0.182	0.999314	257	258-287	287	258-287	260-287	258-281
VVIb01	2	2 349 171	1	7	3	3	5	1	20	2.727	0.743058	287	287-297	297	287-297	287-297	287-294
VVIb23	2	4 864 334	3	5	3	4	5	1	21	1.818	0.874761	286	284-286	284	284-286	284-286	286-290
VMC5g7	2	8 222 941	3	3	2	1	6	0	15	5.273	0.8107	214	195-214	195	195-214	195-214	214
VVIu20	2	16 538 965	4	4	2	1	8	2	21	3.636	0.603815	364	364-387	387	364-387	364-387	364
UDV093	3	1 335 227	3	6	2	1	7	2	21	2,000	0.849145	167	163-167	163	163-167	163	158-167
VMC2e7	3	3 223 011	6	1	1	1	3	5	17	12.727	0.026149	151	151-160	160	151-160	151-154	151-160
VMC1a5	3	5 954 608	2	5	4	0	6	5	22	5.455	0.36346	183	183-202	202	183-202	202	183-202
VVIb59	3	8 121 467	3	5	3	0	6	5	22	4.727	0.450998	356	356-387	387	356-387	355-387	355-356
VVIr46	4	1 079 759	1	6	3	2	5	3	20	1.455	0.918767	380	376-380	376	376-380	376-382	380-385
VMC7h3	4	4 719 044	1	7	3	4	5	2	22	2.364	0.797416	133	131-133	131	131-133	131	133
VrZag21	4	13 648 595	0	8	3	2	8	1	22	6.364	0.272748	199	199-205	205	199-205	201-205	199-205
VVIn75	4	14 823 422	0	8	3	1	9	1	22	8.364	0.13748	190	184-190	184	184-190	184	184-190
VVMD32	4	18 035 848	1	2	5	0	7	1	16	9.455	0.09241	249	236-249	236	236-249	249-270	236-267
VMC6g10	4	23 105 311	2	4	3	2	6	2	19	1.091	0.955	173	164-173	164	164-173	173	164-173
VMC3b9	5	4 472 105	1	6	3	1	5	4	20	2.909	0.7154	99	91-99	91	91-99	91-99	99
VrZag79	5	5 692 460	1	5	4	1	5	5	21	4.727	0.451	247	239-247	239	239-247	239-241	243-247
VVIn33	5	11 299 447	2	6	3	1	4	6	22	5.636	0.343901	282	282-289	289	282-289	282-289	282
VVMC16d4	5	17 185 191	4	4	3	1	5	5	22	4,000	0.549416	151	151-162	162	151-162	NA	151-168
VMC9b5	5	19 752 558	3	4	4	1	6	3	21	2.182	0.82372	241	241-259	259	241-259	241-259	239-241
UDV064	5	22 100 509	4	1	5	5	1	4	20	12.182	0.032403	118	118-133	133	118-133	NA	118-131
VVIp72	6	2 228 251	3	6	1	1	9	1	21	5.636	0.343901	77	77-90	124	77-90	77-90	77
UDV085	6	4 906 509	0	8	3	0	9	0	20	11.636	0.040225	133	124-133	90	124-133	124-131	128-133
VVS5	6	12 956 616	1	7	2	1	7	3	21	3.273	0.658438	99	84-99	84	84-99	84-99	55-99
VVIp28	6	16 922 815	1	7	3	1	7	3	22	3.091	0.686113	258	245-258	245	245-258	245-258	242-258
VVIm43	6	19 003 918	3	3	4	2	7	2	21	2.545	0.770459	96	84-96	84	84-96	84-96	78-96
VrZag62	7	1 766 878	3	6	2	2	8	1	22	2.727	0.743058	195	195-204	204	195-204	195-204	187-195
VVMD6	7	4 186 801	5	5	0	2	8	1	21	7.091	0.214	205	205-208	208	205-208	205-208	199-205
VVIv36	7	8 397 652	4	6	0	1	4	3	18	4.909	0.428206	151	151-157	157	151-157	155-157	151-157
VMC8d11	7	11 699 388	6	4	1	3	3	5	22	8.364	0.13748	136	136-142	142	136-142	136-142	132-136
VVIp75	7	16 569 961	6	4	1	3	4	2	20	6,000	0.306219	238	233-238	233	233-238	233-238	238
VMC1f10	8	292 910	2	3	6	2	7	2	22	6,000	0.306219	190	190-195	195	190-195	195-207	190-207
VVIp04	8	5 145 874	0	4	6	2	6	2	20	7.455	0.189266	99	99-118	118	99-118	96-118	96-99
VVIv15	8	9 604 044	0	4	7	2	7	2	22	10.545	0.061303	78	78-97	97	78-97	94-97	78-92
VMC1b11	8	14 039 124	0	5	6	3	6	2	22	6.909	0.228184	169	169-183	183	169-183	165-183	169-171
VMC3c9	8	17 399 479	0	3	4	1	4	0	12	8.727	0.120767	251	251-258	258	251-258	251-258	251-258
VVIb66	8	18 270 347	0	7	4	1	7	3	22	5.273	0.3838	103	91-103	91	91-103	83-91	83-103
VMC2h10	8	20 817 021	0	7	3	1	7	3	21	4.727	0.450998	105	105-111	111	105-111	105-111	105
VMC1c10	9	563 536	6	4	1	3	5	3	22	5.455	0.36346	154	154-166	166	154-166	143-166	154-166
VVIu37	9	3 971 422	2	2	2	4	4	2	16	3.818	0.577083	226	226-233	233	226-233	226-233	226-233
VMC3g8.2	9	4 420 762	0	5	5	2	5	2	19	5.091	0.404996	165	165-175	175	165-175	165-175	165-175
VMC3h5	9	21 222 733	3	3	0	0	7	1	14	8.182	0.146591	140	140-161	161	140-161	NA	NA
VMC6e4	9	22 675 209	6	5	0	2	7	2	22	7.455	0.189266	173	173-175	175	173-175	NA	165-173
VMC2e11	9	22 676 139	2	6	3	2	7	2	22	1.091	0.954985	128	101-128	101	101-128	93-101	128
VVIh01	10	1 181 256	3	5	3	1	4	6	22	5.455	0.36346	256	241-256	241	241-256	238-241	244-256
Vrzag64	10	1 339 872	2	5	4	2	4	5	22	3.273	0.658438	160	156-160	156	156-160	133-156	136-160
VVIr21	10	1 609 895	3	6	2	1	3	7	22	9.091	0.105528	183	172-183	172	172-183	172-183	166-183
UDV059	10	5 457 979	2	6	3	2	4	5	22	2.727	0.743058	132	132-161	161	132-161	140-161	132-161
VMC8d3	10	5 865 633	1	5	4	2	3	6	21	6.909	0.2281840.	163	163-167	167	163-167	157-167	163-167
UDV063	10	11 895 733	4	2	3	2	6	3	20	3.091	0.686113	125	125-140	140	125-140	140	125-140
UDV016	10	12 198 241	4	3	4	3	5	3	22	2.364	0.797416	165	160-165	160	160-165	160-163	160-165
VVIv37	10	12 614 190	5	2	3	2	2	3	17	6.545	0.257159	161	159-161	159	159-161	156-159	161
VVIp02	11	13 539 428	2	3	2	2	3	4	16	3.455	0.630966	266	266-272	272	266-272	270-272	266
VVMD8	11	19 675 145	3	6	2	4	4	3	22	1.273	0.937988	135	135-138	138	135-138	138-141	135-138
TT251f02	12	291 618	5	3	3	2	6	3	22	3.273	0.658438	190	190-192	192	190-192	NA	190-192
VMC2h4	12	5 447 330	4	5	1	2	6	2	20	2.182	0.82372	234	220-234	220	220-234	220-224	200-234
VVIm11	12	8 350 835	1	5	5	1	8	2	22	5.455	0.36346	280	280-283	283	280-283	280-292	280-283
VMC1g3_2	12	9 973 908	1	5	5	1	8	2	22	5.455	0.36346	125	125-169	169	125-169	125-139	125-169
VMC4f3.1	12	13 067 657	1	3	4	0	9	2	19	8,000	0.156236	172	172-178	178	172-178	172-188	172-178
VVIb10	12	18 063 163	0	2	3	0	7	3	15	8.182	0.146591	74	74-80	80	74-80	72-74	74-80
VMC8g9	12	20 378 485	3	3	0	1	6	1	14	6.182	0.289096	185	166-185	166	166-185	166-171	185-197
VVIn62	13	1 387 838	0	7	3	3	7	1	21	4.905	0.428206	355	355-362	362	355-362	355-362	355-362
VVIm63	13	4 807 861	0	9	0	1	7	2	19	10.474	0.062962	306	299-306	299	299-306	299-306	306
VMC3d12	13	8 083 693	2	6	3	3	7	1	22	1.818	0.874761	199	192-199	192	192-199	NA	192-199
VVIc51	13	8 083 696	3	5	3	3	7	1	22	1.636	0.897598	166	160-166	160	160-166	160-166	160-166
VMC2c7	13	13 864 819	8	1	2	1	4	6	22	19.273	0.001712	152	152-162	162	152-162	152-162	141-152
VMC3b12	13	16 298 014	6	1	2	1	2	5	17	15.235	0.009423	121	121-128	128	121-128	121-128	NA
VVIq32	14	6 641 870	2	5	3	2	8	1	21	2.727	0.743058	101	97-101	97	97-101	97	97-101
VMC2c3	14	20 194 293	3	3	5	3	5	3	22	3.091	0.686113	162	162-167	167	162-167	167-189	162-167
VVMD24	14	24 251 864	4	2	4	4	4	2	20	4.545	0.4745	214	214-217	217	214-217	209-217	214-217
VVIn64	14	26 603 332	3	4	4	3	7	1	22	2.545	0.770459	80	80-82	82	80-82	70-82	80-82
VVIi51	14	28 772 163	3	4	4	3	7	1	22	2.545	0.770459	258	258-262	262	258-262	246-262	258-262
VVIv67	15	10 897 392	3	7	1	3	6	2	22	1.818	0.874761	371	356-371	356	356-371	356-362	362-371
VVIb63	15	11 597 153	6	5	0	4	4	1	20	8.727	0.120767	143	141-143	141	141-143	141-143	143
VVIp33	15	13 060 296	3	8	0	3	6	2	22	4.182	0.523802	401	401-403	403	401-403	399-403	401
VMC5g8	15	14 895 031	2	5	2	1	5	1	16	2.727	0.743058	316	312-316	312	312-316	302-312	312-316
VVIv24	15	15 945 489	2	7	1	2	6	3	21	2,000	0.849145	365	360-365	360	360-365	360	360-365
VMC8g3.2	15	17 666 601	3	6	2	1	8	2	22	2.727	0.743058	280	280-292	292	280-292	290-292	280-292
VVIn52	16	619 647	2	5	2	2	5	3	19	0.727	0.9819	84	84-100	100	84-100	84-100	84-100
VVMD37	16	20 442 394	9	2	0	5	6	0	22	23.818	0.0002361	219	205-219	205	205-219	205-219	219
VVMD5	16	20 785 782	3	6	2	2	4	5	22	2.727	0.743058	228	222-228	222	222-228	222-231	228-235
VMC3c11	17	2 165 152	5	4	2	2	7	2	22	3.273	0.658438	115	115-117	117	115-117	109-115	92-117
VMC9g4	17	8 681 540	2	4	5	2	7	2	22	3.273	0.658438	151	151-163	163	151-163	163	151-167
VVIv08	17	11 011 825	2	4	5	2	7	1	21	4.182	0.523802	367	361-367	361	361-367	361	365-367
VMC2a3	18	948 244	3	5	2	2	6	2	20	0.727	0.981853	157	157-173	173	157-173	157-175	157-173
VVMD17	18	19 873 419	2	5	4	3	8	0	22	4.727	0.450998	217	217-220	220	217-220	217-220	217
VVIn16	18	23 389 686	3	4	4	3	7	1	22	2.545	0.770459	159	153-159	153	153-159	151-153	151-159
VMC7f2	18	26 896 790	3	3	3	3	6	2	20	1.455	0.918767	197	197-201	201	197-201	197-201	197
VMC5h11	19	1 241 284	2	5	3	5	4	2	21	2.727	0.743058	196	190-196	190	190-196	190-196	185-196
VVIp11	19	4 298 291	1	6	4	6	3	2	22	6.909	0.228184	159	149-159	149	149-159	NA	149-152
VVIv70	19	6 325 597	0	5	2	5	0	3	15	10.364	0.065654	185	167-185	167	167-185	167-189	185-196
VVIp31	19	6 697 524	1	2	3	3	2	4	15	6.182	0.289096	178	174-178	174	174-178	174-181	174-178
VVIm03	19	7 078 556	0	7	3	4	3	4	21	5.455	0.36346	369	369-372	372	369-372	369-375	372
VVIp34	19	13 224 073	1	7	3	3	4	4	22	2.545	0.770459	84	84-87	87	84-87	84-87	84-97
VVIv33	19	14 016 375	1	7	3	3	4	4	22	2.545	0.770459	342	342-362	362	342-362	342-362	342
VMC7b1	19	22 955 007	2	6	3	3	4	4	22	1.273	0.937988	242	232-242	232	232-242	232-242	238-242
