# Supplementary material for: A single resistance factor to solve vineyard degeneration due to grapevine fanleaf virus
Source: Commun Biol. 2021 May 28;4:637. doi: 10.1038/s42003-021-02164-4 (PMC8163887; doi:10.1038/s42003-021-02164-4)
Supplement: Supplementary file 7 — Reporting Summary [file 42003_2021_2164_MOESM7_ESM.pdf]

nature research

Corresponding author(s): Didier Merdinoglu

Last updated by author(s): Apr 23, 2021

Reporting Summary

Nature Research wishes to improve the reproducibility of the work that we publish. This form provides structure for consistency and transparency in reporting. For further information on Nature Research policies, see our Editorial Policies and the Editorial Policy Checklist.

Statistics

For all statistical analyses, confirm that the following items are present in the figure legend, table legend, main text, or Methods section.

n/a	Confirmed	
☐	☒	The exact sample size (n) for each experimental group/condition, given as a discrete number and unit of measurement
☐	☒	A statement on whether measurements were taken from distinct samples or whether the same sample was measured repeatedly
☐	☒	The statistical test(s) used AND whether they are one- or two-sided<br>*Only common tests should be described solely by name; describe more complex techniques in the Methods section.*
☒	☐	A description of all covariates tested
☒	☐	A description of any assumptions or corrections, such as tests of normality and adjustment for multiple comparisons
☒	☐	A full description of the statistical parameters including central tendency (e.g. means) or other basic estimates (e.g. regression coefficient) AND variation (e.g. standard deviation) or associated estimates of uncertainty (e.g. confidence intervals)
☐	☒	For null hypothesis testing, the test statistic (e.g. F, t, r) with confidence intervals, effect sizes, degrees of freedom and P value noted<br>*Give P values as exact values whenever suitable.*
☒	☐	For Bayesian analysis, information on the choice of priors and Markov chain Monte Carlo settings
☒	☐	For hierarchical and complex designs, identification of the appropriate level for tests and full reporting of outcomes
☒	☐	Estimates of effect sizes (e.g. Cohen's d, Pearson's r), indicating how they were calculated

Our web collection on statistics for biologists contains articles on many of the points above.

Software and code

Policy information about availability of computer code

Data collection	3500 Series Data Collection V2 – LifeTechnologies Ref 4475161 - Software for the data collection from the ABI 3500 genetic analyser
Data analysis	GeneMapper V5 - LifeTechnologies Ref 4475038 - Software for fragment analysis, sizing and allele detection<br>JoinMap 3.0 - Kyazma - Software for the calculation of genetic linkage maps in experimental populations

For manuscripts utilizing custom algorithms or software that are central to the research but not yet described in published literature, software must be made available to editors and reviewers. We strongly encourage code deposition in a community repository (e.g. GitHub). See the Nature Research guidelines for submitting code & software for further information.

Data

Policy information about availability of data

All manuscripts must include a data availability statement. This statement should provide the following information, where applicable:

- Accession codes, unique identifiers, or web links for publicly available datasets
- A list of figures that have associated raw data
- A description of any restrictions on data availability

The authors declare that the data supporting the findings of this study are available within the paper and its supplementary information files.

Field-specific reporting

Please select the one below that is the best fit for your research. If you are not sure, read the appropriate sections before making your selection.

- [x] Life sciences
- [ ] Behavioural & social sciences
- [ ] Ecological, evolutionary & environmental sciences

For a reference copy of the document with all sections, see nature.com/documents/nr-reporting-summary-flat.pdf

Life sciences study design

All studies must disclose on these points even when the disclosure is negative.

Sample size	When the experiments were performed on plants from cutting, 5 to 15 biological replicates were used per modality (genotype). The number of replicates was mainly determined by the availability of plants, the total size of each experiment and our own expertise in the mesure of the observed phenotypes
Data exclusions	No data were excluded from the analyses
Replication	When possible, we used biological replicates (cuttings) to ensure the significance of the experimental findings. If this was not the case, we carried out multi-year measurements. Details by experiments are given in the main text or the legend of the figures or tables.
Randomization	When the use of biological replicates were not possible (results presented in Figure 4 and Supplementary Data 1), we used a total randomisation for the distribution of individuals in the greenhouse. No structure was observed between the spatial distribution of the plants displaying different phenotypes
Blinding	In most of the experiments, the modalities (genotypes) were encoded. Furthemore, for genetic analyses, the investigators responsible for genotyping were different from those responsible for phenotyping, the convergence of the data being achieved only at the time of the final analysis.

Reporting for specific materials, systems and methods

We require information from authors about some types of materials, experimental systems and methods used in many studies. Here, indicate whether each material, system or method listed is relevant to your study. If you are not sure if a list item applies to your research, read the appropriate section before selecting a response.

Materials & experimental systems

n/a	Involved in the study
[ ]	[x] Antibodies
[x]	[ ] Eukaryotic cell lines
[x]	[ ] Palaeontology and archaeology
[x]	[ ] Animals and other organisms
[x]	[ ] Human research participants
[x]	[ ] Clinical data
[x]	[ ] Dual use research of concern

Methods

n/a	Involved in the study
[x]	[ ] ChIP-seq
[x]	[ ] Flow cytometry
[x]	[ ] MRI-based neuroimaging

Antibodies

Antibodies used	Reagents used for GFLV detection :<br>Coating IgG- Ref : 120412<br>Conjugate : Ref : 120422<br>Supplier: BIOREBA AG<br>Christoph Merian-Ring 7<br>CH-4153 Reinach BL1<br>Switzerland<br>Site web: https://www.bioreba.ch/saas/web/bioreba/
Validation	Quality control datasheet and lot number available
